# Effects of unilateral and bilateral training on performance in team sports athletes: a systematic review and meta-analysis

**DOI:** 10.5114/biolsport.2026.159564

**Published:** 2026-03-16

**Authors:** Bitai Wu, Mingyue Yin, Zhiquan Song, Meiling Tao, Kai Xu, George P. Nassis, Chris Bishop, Olivier Girard

**Affiliations:** 1 School of Physical Education and Sports Science, Hengyang Normal University, Hengyang, Hunan, China; 2 School of Coaching, Shanghai University of Sport, Shanghai, China; 3 School of Physical Education, University of Science and Technology Beijing, Beijing, China; 4 College of Sport Science, University of Kalba, Sharjah 89841, United Arab Emirates; 5 School of Science and Technology, London Sport Institute, Middlesex University, London, UK; 6 School of Human Science (Exercise and Sport Science), University of Western Australia, Perth 6009, Australia

**Keywords:** Unilateral training, Bilateral exercise, Strength development, Jumping, Sprinting, Agility, Neuromuscular adaptation, Team sports

## Abstract

Team sports athletes rely heavily on unilateral movements, yet the comparative performance benefits of unilateral training (UT) versus bilateral training (BT) remain controversial. This meta-analysis systematically examined the effects of UT and BT on strength, jumping, sprinting, and agility in team sports, while considering sport type and training program as potential moderators. A systematic search of PubMed, Web of Science, Cochrane Library, and CNKI databases was conducted to identify controlled trials meeting the PICOS criteria. Primary outcomes were strength, jumping (countermovement jump [CMJ], horizontal jump [HJ], reactive strength index [RSI]), sprinting, and agility. Pooled effects were calculated using standardized mean difference (Hedges’ g) through a three-level meta-analysis model, with subgroup and moderator analyses conducted. Fifteen studies involving 355 team sports athletes (basketball, soccer, rugby, and ice hockey) were included. The meta-analysis showed no significant differences between UT and BT in overall strength, jumping, sprinting, and agility. However, UT significantly outperformed BT in unilateral strength (g = 0.68, p = 0.007), unilateral CMJ (g = 0.37, p = 0.025), and unilateral HJ (g = 0.45, p = 0.03). No significant differences were found in bilateral strength, bilateral jumping, and RSI. Subgroup analysis revealed no overall differences across sports, but in basketball, UT had significant advantages over BT in sprinting (g = -0.37, p = 0.04) and agility (g = -0.77, p = 0.04). Regarding training types, UT using plyometric training outperformed BT in strength (g = 0.54, p = 0.03) and sprinting (g = -0.30, p = 0.03), while UT with compound training had significant advantages in agility (g = -0.74, p = 0.04). UT outperforms BT in improving unilateral strength and jumping ability, while producing comparable effects in bilateral performance, sprinting, and agility. These findings underscore the value of UT in team sports involving unilateral movements. Future research should explore its long-term effects, recovery from injury, and neuromuscular adaptations to better optimize training.

## KEY POINTS

1. For team sports athletes, UT significantly enhances unilateral strength and jumping performance, while producing effects comparable to BT on bilateral strength, bilateral jumping, reactive strength index, agility, and sprinting.
2. Sport type and training interventions influence outcomes. In basketball, UT produces greater improvements in agility and sprint performance.
3. Regarding training interventions, UT combined with plyometric exercises significantly improves strength and sprint performance, while UT with compound training enhances agility.

## INTRODUCTION

Team sports such as basketball, football, handball, rugby, volleyball, and ice hockey rely heavily on unilateral lower-limb actions during play [1–4]. These include single-leg braking and reacceleration, rapid direction changes, short sprints with abrupt starts and stops, single-leg takeoffs and landings, as well as striding and lateral movements. Lower-limb performance is crucial for effective skill execution, as leg strength and explosive power strongly correlate with sprinting and agility [5–7]. However, frequent unilateral movements may actually exacerbate the occurrence of the asymmetry phenomenon, reducing lower-limb asymmetry might enhance overall function and sport-specific performance [8, 9] and lower injury risk [10].

Unilateral training (UT) involves single-limb exercises [11] and is often combined with bilateral training (BT) to enhance movement specificity [12]. Overall, UT improves neuromuscular coordination by activating synergistic and core stabilizing muscles, enabling cross-education of the contralateral limb, correcting bilateral limb deficits (BLD), and promoting sport-specific adaptations [13–19]. Studies consistently show that UT improves strength, jumping, sprinting, and balance [20–23], with clear benefits for team sports that require frequent unilateral direction changes, multidirectional speed, rapid braking, and lateral jumps. Due to movement specificity, muscle recruitment and training effects vary accordingly [23]. Following training specificity principles, UT transfers more readily to unilateral performance, while BT better supports bipedal performance [13].

Research has clarified UT and BT mechanisms from both neural and mechanical perspectives. First, the cross-education effect shows unilateral resistance training enhances strength in the untrained limb via central adaptations such as increased neural drive and motor unit discharge rate [16, 24, 25]. Second, the BLD occurs when simultaneous limb contractions produce less force than the sum of individual efforts, due to bihemispheric inhibition and coordinated motor control [17]. Cross-education is particularly pronounced during eccentric training, providing a strong physiological rationale for applying UT in both rehabilitation and competitive contexts [26].

Despite growing research, evidence on UT and BT effects on athletic performance remains limited [23, 27–31]. First, previous reviews and meta-analyses often pooled data from general populations and diverse sports, offering little insight specific to team sports athletes, who engage in high-contact, intermittent, high-intensity activities dominated by unilateral movements. Second, training protocols vary widely in exercise type, intensity, and volume, program duration, and outcomes, leading to inconsistent conclusions about moderators such as sport type and training design. Third, most evaluations focus on jumping and strength, with comparatively fewer comprehensive assessments of sprinting and agility. Accordingly, clarifying the distinct effects of UT and BT on lower-limb performance in team sports athletes is essential for optimizing training and enhancing competitive outcomes.

This meta-analysis aimed to compare UT and BT on lower-limb performance in team sports athletes, specifically i) strength, jumping, sprinting, and agility; ii) moderating factors such as sport type and training protocols; and iii) optimal strategies to guide evidencebased practice.

## MATERIALS AND METHODS

The systematic review adhered to the 2020 Preferred Reporting Items for Systematic Reviews and Meta-Analyses (PRISMA) guidelines [32]. The completed PRISMA 2020 checklist is available in Electronic Supplementary Material Appendix S1. Additionally, this review has been registered in the OSF database, https://doi.org/10.17605/OSF.IO/S8CMA.

### Information Sources

Database searches were conducted in PubMed, Web of Science (Core Collection), Cochrane Library (Embase, CT.gov, and ICTRP), and CNKI. Eligible studies were full-text articles with no restrictions on publication date, sample, or language, provided the title and abstract were in English and/or Chinese. Three systematic snowballing searches were applied: 1) checking reference lists of included articles; 2) reviewing articles citing the included articles; 3) exploring “similar articles” or “find similar” (MEDLINE, Embase). Searches were conducted on February 6, 2025, and updated on August 17, 2025.

### Search Strategy

The search formula was developed based on a previous review of similar systems [33]. The following syntax was used: (“Unilateral exercises” OR “Unilateral resistance training” OR “Single leg training” OR “Unilateral limb exercises”) AND (“Bilateral exercises” OR “Bilateral resistance training” OR “Bilateral limb exercises”) AND (“Unilateral and Bilateral resistance training” OR “Unilateral and Bilateral plyometric training” OR “Unilateral and Bilateral Composite/Complex/ Combine training” OR “Unilateral and Bilateral flywheel training”) AND (“Jump of ability” OR “Ability of sprint” OR “Maximum force” OR “Ability of change of direction” OR “Ability of Balance”) AND (“Basketball players” OR “Soccer players” OR “Rugby players” OR “Volleyball players” OR “Handball players” OR “Hockey players”). Additionally, PROSPERO and the Cochrane Database of Systematic Reviews were searched for previously published protocols. The complete search strategies and results for other databases are provided in Electronic Supplementary Material Appendix S2.

### Selection Process

Retrieved records were manually deduplicated by an independent reviewer (BTW) using EndNote X9 [Clarivate Analytics, 2018]. The cleaned dataset was then screened by two independent researchers (BTW and YYX) for titles and abstracts based on predefined inclusion and exclusion criteria. Disagreements were resolved by a third independent researcher (MYY). The same two researchers reviewed the full texts for final inclusion. Intrarater reliability was high (Cohen’s kappa κ = 0.83), indicating *almost perfect* agreement [34]. Remaining discrepancies were resolved through discussion following the established protocol. Additionally, relevant studies were identified through reference lists of previous systematic reviews and the expertise of the research team to capture articles not retrieved in the initial search.

### Eligibility Criteria

*A priori* inclusion and exclusion criteria were applied using the PICOS framework. Eligible studies included human participants of any sex, age, or training status, as defined by McKay et al [35]. Studies involving animal subjects or participants with chronic diseases were excluded at the title/abstract screening stage.

Interventions required supervised UT with no limitations on mode (i.e., intensity, volume, or type) and a duration of ≥ 4 weeks. Comparator groups included BT only, ensuring consistency with the protocol variables.

Eligible outcomes had to include at least one physiological adaptation or performance measure. Physiological adaptations covered muscle strength (i.e., isometric, isokinetic, maximal voluntary contraction, and 1RM). Performance outcomes included countermovement jump (CMJ), horizontal jump (HJ), reactive strength index (RSI), sprint speed/time, and agility. Studies focusing only on molecularlevel physiological processes were excluded.

Only original research with between-group controlled trial designs (parallel or crossover, randomized or not) were included. Acute studies, reviews, opinions/viewpoints articles, validation studies, books and case studies were excluded.

### Data Extraction

Data extraction was conducted by two reviewers (BTW and YYX) utilizing a customized Excel extraction worksheet finalized before the full-text review. They independently extracted author information, study characteristics, participant demographics, training protocols, and outcomes. Inter-reviewer consistency was evaluated using Pearson correlation coefficients (*r*), with an initial overall mean *r* = 0.86, indicating strong agreement [36]. Discrepancies were resolved through discussion, with no need for third-party arbitration (MYY). If data were missing or presented only in graphical form, study authors were contacted. When unavailable, values were extracted using WebPlot-Digitizer 4.1 (https://automeris.io/WebPlotDigitizer) [37]. Studies with unresolved missing data were excluded from the final analysis. For each group, the mean, standard deviation (SD), and sample size were extracted pre- and post-intervention.

### Risk of Bias and Quality of Methods Assessment

Risk of bias was assessed using the Cochrane Collaboration’s Risk of Bias tool 2 (Rob2) [38], which evaluates random sequence generation, random allocation concealment, blinding of outcome assessment, incomplete outcome data, and selective outcome reporting. Disagreements were resolved through discussion or, when needed, by a third reviewer. For non-randomized studies, the Cochrane’s Risk of Bias In Non-Randomized Studies of Interventions (ROBINS-I) [39] was applied, assessing bias across seven domains: confounding, participant selection, intervention categorization, adherence to intended interventions, handling of missing data, outcome measurement, and selection of reported results. Additionally, the physiotherapy evidence database (PEDro) [40] scale was used to assess methodological quality. The PEDro scale rates studies from 0–10, with scores ≥ 6 considered high quality, 4–5 moderate quality, and ≤ 3 low quality.

### Statistical Analysis

#### Data Synthesis and Effect Measures

We extracted the mean, SD, and sample size reported for each group pre- and post-intervention. Because the included studies assessed outcomes with different measurement tools, or reported different outcomes, we calculated the standardized mean difference (SMD) as the effect size measure, as opposed to the raw mean difference, to allow for the pooling of results across studies. We pooled effects using pre- and post-intervention differences (M ± SD) for each outcome indicator. The mean difference (M_change_) and SD of the change (SD_change_) were calculated using the following formulae [41–43]. Furthermore, mean change represents the change from pre- to post-test in the UT condition minus the change from pre- to post-test in the BT condition. The first step involved calculating the difference in means:

$${\text{M}}_{\text{change}}={\text{M}}_{\text{post}}-{\text{M}}_{\text{pre}}$$ (1)

where M_change_ is the raw mean difference, M_post_ is the reported mean post-intervention, and M_pre_ is the reported mean pre-intervention [44].

Then, the SD of the change in means (SD_change_) was calculated as follows [44]:

$${\text{SD}}_{\text{change}}=\sqrt{{\text{SD}}_{\text{pre}}^{2}+{\text{SD}}_{\text{post}}^{2}-(2\times r\times {\text{SD}}_{\text{pre}}\times {\text{SD}}_{\text{post}})}$$ (2)

where SD_change_ is the standard deviation of the difference in means, SD_pre_ is the standard deviation from pre-intervention, and SD_post_ is the standard deviation from post-intervention, and *r* is the correlation coefficient [44]. While using the pooled SD of baseline scores is a simpler alternative, both SD_change_ and pooled SD of baseline scores are recommended [45], depending on the research question [43, 46–48]. We ultimately selected SD_change_ as the primary method, based on our meta-analysis objectives and comparative results from both methods (Electronic Supplementary Material Appendix S3).

Considering the relatively small sample sizes in most included studies, Hedge’s g (g) was used as the mean effect size point estimate in each analysis, it must be pointed out that the positive scores indicate outcomes favoring UT, except for agility and sprint tests, where negative scores indicate greater reductions in time and therefore greater performance improvements, meaning negative ES(g) favor UT, using the following formula [49]:

$$\begin{array}{l} \;\text{Hedge’s}g=\\ \frac{\left(\text{UT}[{\text{M}}_{\text{change}}]-\text{BT}[{\text{M}}_{\text{change}}]\right)}{{\text{SD}}_{\text{change}}}\times (1-\frac{3}{4({\text{n}}_{1}+{\text{n}}_{2}-2)-1}) \end{array}$$ (3)

where M_change_ is the mean difference between the UT and BT alone groups; *n*_1_ and *n*_2_ are the sample sizes of these two groups; and SD_pooled_ is the pooled standard deviation of the measurements [49]. The specific formulae are as follows:

$${\text{SD}}_{\text{pooled}}=\sqrt{\frac{\left((n_{1}-1)\times SD_{1}^{2}+(n_{2}-1)\times SD_{2}^{2}\right)}{\left(n_{1}+n_{2}-2\right)}}$$ (4)

where *n*_1_ and *n*_2_ are the sample sizes of the two groups, and SD_1_ and SD_2_ are the standard deviations of these groups. *g* were classified as *trivial* (0.2), *small* (0.2–0.5), *medium* (0.5–0.8), and *large* (> 0.8) [50].

#### Meta-Analysis and Heterogeneity

A three-level meta-analysis was conducted using the generic inversevariance pooling method to aggregate Hedge’s g, conducted with the meta and metafor packages in the statistical software R (V.4.2.0) [51]. The DerSimonian-Laird random-effects model [52] was applied to account for between-study heterogeneity. This model assumes that effect sizes are derived from a distribution of true effects, rather than a single homogeneous population. Given the variation in study designs, interventions, and populations, the random-effects model incorporates heterogeneity [44] by assuming that underlying effects follow a normal distribution, leading to a more accurate estimation of the overall effect size [53].

In studies reporting nested or multiple effect sizes, these were often correlated. Including all effect sizes simultaneously could violate the independence assumption of traditional meta-analyses [54], while considering only one would be overly conservative and risk underestimating the true effect [55]. To address this, we applied a three-level meta-analysis following the methods of Assink & Wibbelink [56]. This approach decomposes variance into sampling variance (level 1), within-study variance (level 2), and between-study variance (level 3), thereby accounting for correlated and hierarchical effects [57]. By retaining multiple effect sizes per study, the three-level meta-analysis enhances statistical power and provides a more accurate estimate of effect sizes [56]. Parameters were estimated using the restricted maximum likelihood method, and results were cross-checked with the maximum likelihood method to ensure stability.

Tests of individual coefficients and confidence intervals (CI) were based on the t-distribution [58]. A prediction interval (PI) was also computed using the t-distribution to estimate the treatment effect while accounting for heterogeneity, providing additional information beyond the CI, especially considering the use of a random-effects model [59, 60]. Studies were identified as statistical outliers when their CI did not overlap with the pooled-effect CI. Influence analysis was performed using the leave-one-out method to assess the effect of individual studies. While several metrics can assess heterogeneity (e.g., Cochrane’s Q, I^2^ statistic, tau^2^), the I^2^ statistic is most commonly used and recommended [61]. Therefore, the main analysis reports I2, interpreted as follows: 0%-25%, might not be important; 25%-50%, moderate heterogeneity; 50%-75%, substantial heterogeneity; and 75%-100%, considerable heterogeneity [44].

Additionally, the statistical power of the primary pooled effect was calculated to account for the possibility of false negatives due to insufficient statistical power. Power calculations were performed using the metameta package [62].

#### Subgroup and Meta-regression Analysis

To explore sources of heterogeneity and potential moderating factors, subgroup analyses were conducted for categorical and continuous variables, respectively. In line with published recommendations, subgroup analyses were restricted to subgroups with ≥ 5 studies to avoid unstable estimates and very low statistical power. The main categorical moderators examined were sport discipline and training type. Subgroup analyses were implemented within a three-level random-effects framework, with random intercepts for study and for effect sizes nested within studies. Standardized mean differences (yi) and their sampling variances (vi or V) were modeled using restricted maximum likelihood (REML), with test statistics based on a t-distribution [51].

#### Risk of Publication Bias and Sensitivity Analysis

Publication bias was assessed using a contour-enhanced funnel plot [63] and Egger’s asymmetry test [64, 65]. Tests were performed only when k ≥ 5 [66], with p > 0.05 indicating no evidence of publication bias. Funnel plots and Egger’s regression tests evaluate the symmetry of effect size, either through subjective or quantitative measures, thereby assessing the risk of publication bias in the included studies.

Additionally, in the sensitivity analysis for the selection of the correlation coefficient (r), we systematically examined the assumed correlation coefficient required to construct the covariance matrix for the effect sizes. Since the true value of this parameter is unknown, we tested five values within a plausible range of 0.5 to 0.9 (r = 0.5, 0.6, 0.7, 0.8, 0.9). For each value of r, the corresponding covariance matrix was constructed using the impute_covariance_matrix function, and a three-level random-effects model (rma.mv) was fitted. Based on the principle of statistical stability, we calculated the absolute deviation of each model’s overall effect size estimate from the median of these estimates. The correlation coefficient corresponding to the smallest absolute deviation was ultimately selected as the standard parameter for the primary analysis, ensuring the robustness of the results to variations in the assumed correlation.

Our sensitivity analysis was conducted on three levels. First, we compared methods for selecting standard deviations. The primary analysis utilized the standard deviation of the difference between pre- and post-measurements, while the secondary analysis employed the baseline standard deviation for comparison. Second, we conducted a leave-one-out analysis, sequentially removing each study to assess its influence on the pooled effect. Lastly, we identified potential outliers using the three-level meta-analysis and examined their influence on the overall effect size in the primary model. Specifically, Cook’s distance [67] and studentized residuals [68] were employed to diagnose leverage, outliers, and influential cases at the within-study (level 2) and between-study (level 3) levels. Cases were flagged if Hat and Cook’s distance values exceeded three times the means, or if studentized residuals were > 3. The three-level meta-analysis was then repeated with these outliers excluded to assess model stability.

#### Certainty of the Evidence

The risk of bias was considered in interpreting results using the Grading of Recommendations Assessment, Development, and Evaluation (GRADE) methodology, which rates evidence certainty as “high”, “moderate”, “low” or “very low” [69]. GRADE assessments were completed by one reviewer (BTW) and independently checked by a second (MYY). The overall quality was initially rated as high and downgraded one level to moderate, low, or very low for each of the following limitations [70, 71]: > 50% of studies in the metaanalysis had one or more risk-of-bias items assessed to be high risk the certainty was downgraded by one level for risk of bias; if substantial statistical heterogeneity was present (I^2^ > 50%), the certainty was downgraded by one level for inconsistency; if the 95% CI of the pooled estimate crossed a decision-relevant threshold (e.g., if the CI included the possibility of a trivial effect (i.e., g < 0.2) while the mean effect was small, or the CI included a large standardized mean difference while the mean standardized mean difference was moderate) the certainty was downgraded the certainty was downgraded by one level for imprecision; and if publication bias was strongly suspected either by visual inspection of the funnel plot or by Egger’s test (p < 0.05) when the number of included studies was ≥ 10, the certainty was downgraded by one level for publication bias.

## RESULTS

### Studies Retrieved

**FIG. 1:**
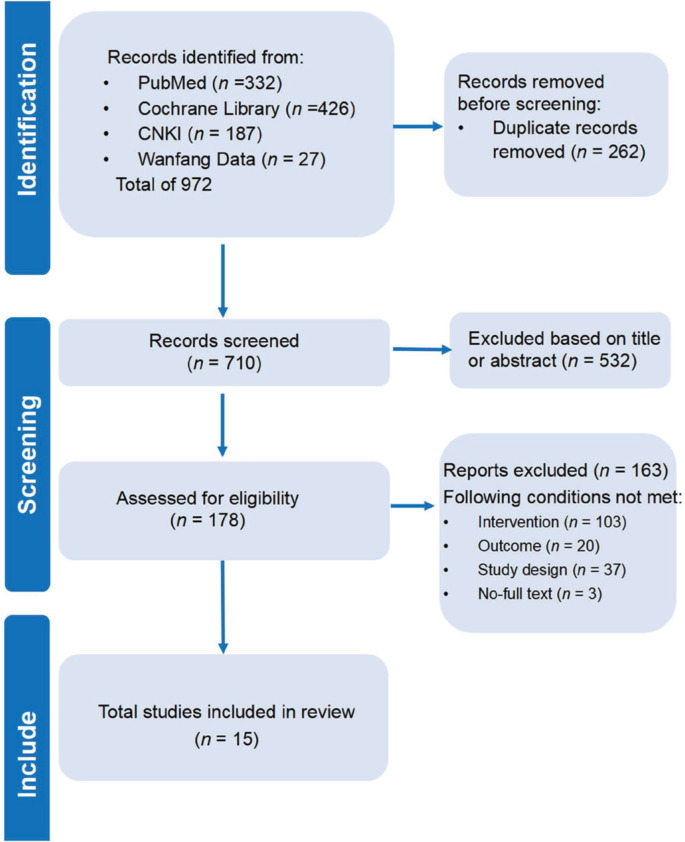
PRISMA flow diagram of study selection.

The initial search yielded 972 publications: 820 from the primary search, 150 from an updated search conducted six months later, and two from other sources. After screening, 15 studies met the inclusion criteria [21, 72–85] and were included in the meta-analysis (Fig. 1).

### Characteristics of Included Studies

All 15 studies were randomized controlled trials, involving 355 participants (337 males and 18 participants with unspecified sex). Sample sizes ranged from 10 to 75, and intervention durations from 4 to 18 weeks. By sport, studies included basketball (7 studies, 176 participants) [21, 73–78], soccer (3 studies, 87 participants) [79–81], rugby (4 studies, 74 participants) [82, 83, 86, 85], and hockey (1 study, 18 participants) [72]. Intervention types included complex training (5 studies, 98 participants) [21, 77, 79, 80, 85], resistance training (5 studies, 87 participants) [72, 78, 82–84], and plyometric training (5 studies, 170 participants) [73–76, 81]. More details on participants and intervention programs are presented in Table 1.

**TABLE 1:** Characteristics for the studies included in the meta-analysis

Study	Participants						Outcome measure

Author, year	Sex	Identity	N	Age (years)	Height (cm)	Weight (kg)	performance
Zhaoqing [73], 2021	M	B	UT(10)	20.6 ± 1.5	185.6 ± 3.6	79.9 ± 6.5	CMJ, RSI, COD, V-cut
			BT(10)	20.4 ± 1.3	186.1 ± 4.0	80.7 ± 5.0	

YongXing [74], 2024	M	B	UT(25)	16.2 ± 0.6	175.1 ± 7.1	61.9 ± 6.9	Peak landing forces
			BT(25)	16.4 ± 0.6	180.6 ± 7.1	69.6 ± 8.5	

Bogdan Belegišanin [75], 2025	M	B	UT(11)	15.5 ± 0.5	186.0 ± 6.0	71.0 ± 12.0	Maximum force, 5-0-5 test, CMJ, RSI, Sprint
			BT(11)	15.2 ± 0.4	183.0 ± 4.0	69.0 ± 7.0	

JianChun [76], 2024	M	B	UT(16)	15.9 ± 0.9	175.5 ± 6.9	62.4 ± 5.2	Maximum force, CMJ, 5-0-5 test
			BT(16)	16.3 ± 0.8	180.5 ± 5.0	68.9 ± 6.7	

Gonzalo-Skok [21], 2017	M	B	UT(11)	16.8 ± 1.7	190.4 ± 6.9	76.9 ± 8.6	Maximum force, CMJ, COD, Sprint
			BT(11)	16.7 ± 1.7	188.9 ± 7.5	74.9 ± 9.6	

Tianyu [77], 2024	M	B	UT(10)	19.9 ± 1.4	183.2 ± 6.8	77.9 ± 5.9	Maximum force, CMJ, Sprint, 5-0-5 test, T-test
			BT(10)	20.8 ± 1.1	183.0 ± 5.8	76.2 ± 8.7	

Hernández-Davó [78], 2018	M	B	UT(5)	21.6 ± 2.4	180.0 ± 0.9	83.6 ± 21.4	Maximum force, CMJ, Standing triple jump, T-test
			BT(5)				

Stern [79], 2020	M	S	UT(11)	17.6 ± 1.2	179.6 ± 7.2	77.3 ± 7.9	Maximum force, CMJ, DJ, Standing horizontal jump, Sprint, 5-0-5 test
			BT(12)				

Ramirez-Campillo [80], 2018	M	S	UT(9)	17.3 ± 1.1	177.1 ± 5.9	64.9 ± 5.5	Maximum force, CMJ, COD, Standing horizontal jump
			BT(9)	17.6 ± 0.5	174.9 ± 5.3	68.3 ± 3.6	

Drouzas [81], 2020	M	S	UT(23)	9.9 ± 1.8	142.2 ± 8.7	39.3 ± 8.2	Maximum force, CMJ, SJ, Horizontal Jump, Sprint, COD
			BT(23)	10.0 ± 0.5	139.2 ± 7.0	36.1 ± 7.8	

Xiang [82], 2023	M	R	UT(9)	15.3 ± 0.3	178.9 ± 8.2	74.3 ± 7.7	Maximum force, CMJ, Sprint
			BT(9)	15.2 ± 0.5	180.0 ± 4.5	74.2 ± 6.5	

Appleby.B [83], 2020	M	R	UT(10)	23.1 ± 4.1	186.3 ± 5.1	104.6 ± 11.5	Sprint, COD
			BT(13)	21.8 ± 3.3	184.3 ± 5.9	101.3 ± 12.8	

Speirs [84], 2016	Non	R	UT(9)	18.1 ± 0.5	183.0 ± 3.4	96.7 ± 9.3	Maximum force, Sprint, COD
			BT(9)	18.1 ± 0.5	185.0 ± 8.9	98.1 ± 13.4	

Fisher [85], 2014	M	R	UT(8)	19.8 ± 1.4	182.0 ± 0.8	82.6 ± 6.5	T-test, Illinois agility test, Sprint
			BT(7)	20.1 ± 1.7	180.0 ± 0.6	85.7 ± 7.0	

Boxuan [72], 2020	M	H	UT(9)	15.7 ± 0.6	174.6 ± 3.9	64.5 ± 4.6	Sprint, Standing Triple Jump
			BT(9)	15.6 ± 0.9	171.7 ± 2.4	62.7 ± 10.8	

**Study**	**Training program**						

**Author, year**	**Weeks**	**Time/Week**	**Type**	**Intensity**	**Exercises**	**Sets**	**Reps**

Zhaoqing [73], 2021	8	2	PT	maximal effort	Unilateral vertical, horizontal, lateral plyometric	6	4 reps/side
					Bilateral vertical, horizontal, lateral plyometric		8 reps

YongXing [74], 2024	8	2	PT	maximal effort	Unilateral plyometric	1	50–75 reps
					Bilateral plyometric		

Bogdan Belegišanin [75], 2025	6	2	PT	$\frac{0.005\text{kg}/{\text{m}}^{2}}{0.010\text{kg}/{\text{m}}^{2}}$	Split squat	2–3	8 reps
					Half squat		

JianChun [76], 2024	8	2	PT	maximal effort	Unilateral vertical, horizontal plyometric	2	2+5 reps/side
					Bilateral vertical, horizontal plyometric		4+10 reps

Gonzalo-Skok [21], 2017	6	2	CT	80–100% 1RM	Unilateral squat+Single DJ+Single CMJ	4	Non
					Squat+DJ+CMJ		

Tianyu [77], 2024	8	2	CT	85%1RM+ self-weight	Bulgarian squat+Split jump	5	6+10 reps
					Squat+jump		

Hernández-Davó [78], 2018	6	2	RT	0.025 kg/m2	Unilateral squat	4	4 reps/side
					squat		8 reps

Stern [79], 2020	6	2	CT	75–85% 1RM	Elevated split squat+Unilateral DJ, Horizontal jump, CMJ	4	3–6 reps/side
					Squat+DJ+Horizontal jump+CMJ		3–6 reps

Ramirez-Campillo [80], 2018	8	2	CT	70%1RM 10–25 cm	Unilateral knee extensors, flexors+Unilateral DJ, horizontal jump	3–5	3–10 reps/side
					Knee extensors, flexors+DJ+Horizontal jump		3–10 reps

Drouzas [81], 2020	10	2	PT	maximal effort	Unilateral vertical, horizontal, lateral plyometric	3–5	50% of the BLT
					Bilateral vertical, horizontal, lateral plyometric		6–10 reps

Xiang [82], 2023	5	2	RT	70–90% 1RM	Unilateral leg press	1	2–7 reps
					Bilateral leg pres		

Appleby.B [83], 2020	12	2	RT	45–85% 1RM	Unilateral resistance	2–3	4–8 reps
					Bilateral resistance		

Speirs [84], 2016	5	2	RT	75–92% 1RM	Single elevated squat	4	3–6 reps
					Squat		

Fisher [85], 2014	6	2	CT	80%1RM	Unilateral leg squat+Unilateral horizontal, lateral jump+Max speed jumps+Unilateral box jump	1–3	6 reps/side
					Squat+horizontal, lateral jump+Max speed jumps+ Box jump		6 reps

Boxuan [72], 2020	8	2	RT	87%1RM	Bulgarian squat	5	5 reps
					Squat		

Note: N, sample size; PT, plyometric training; RT, resistance training; CT, complex training; CMJ, countermovement jump; RSI, reactive strength index; COD, change of direction; DJ, drop jump; B, basketball; S, soccer; R, rugby; H, hockey.

### Primary Analysis

**FIG. 2:**
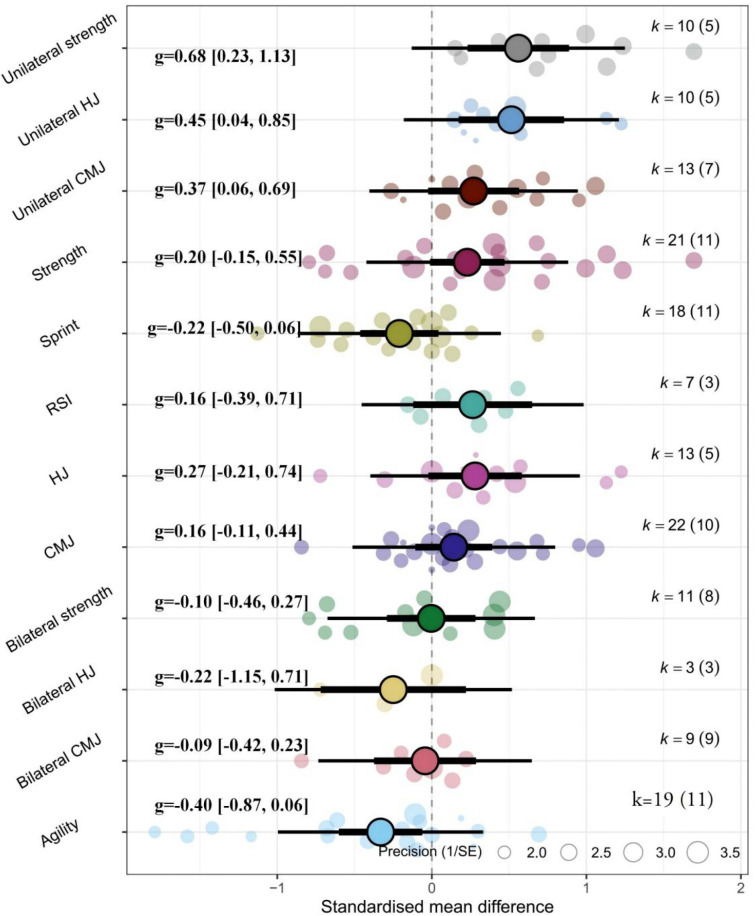
Primary pooled effect sizes for the outcomes. note: Standardized mean differences (SMD) with 95% confidence intervals for various performance outcomes comparing unilateral and bilateral training interventions. Each row represents a separate meta-analysis for a specific outcome variable. k represents the number of included study effect size comparisons, with the number in parentheses indicating the number of unique studies contributing to those comparisons. The effect size (ES) is reported as Hedges’ g with its corresponding 95% confidence interval. In addition to agility and sprint testing, positive values indicate results that favor unilateral training. Circle size is scaled by precision (1/standard error), with larger circles representing more precise estimates.

The summary forest plots of all outcome measures are shown in Figure 2.

**FIG. 3:**
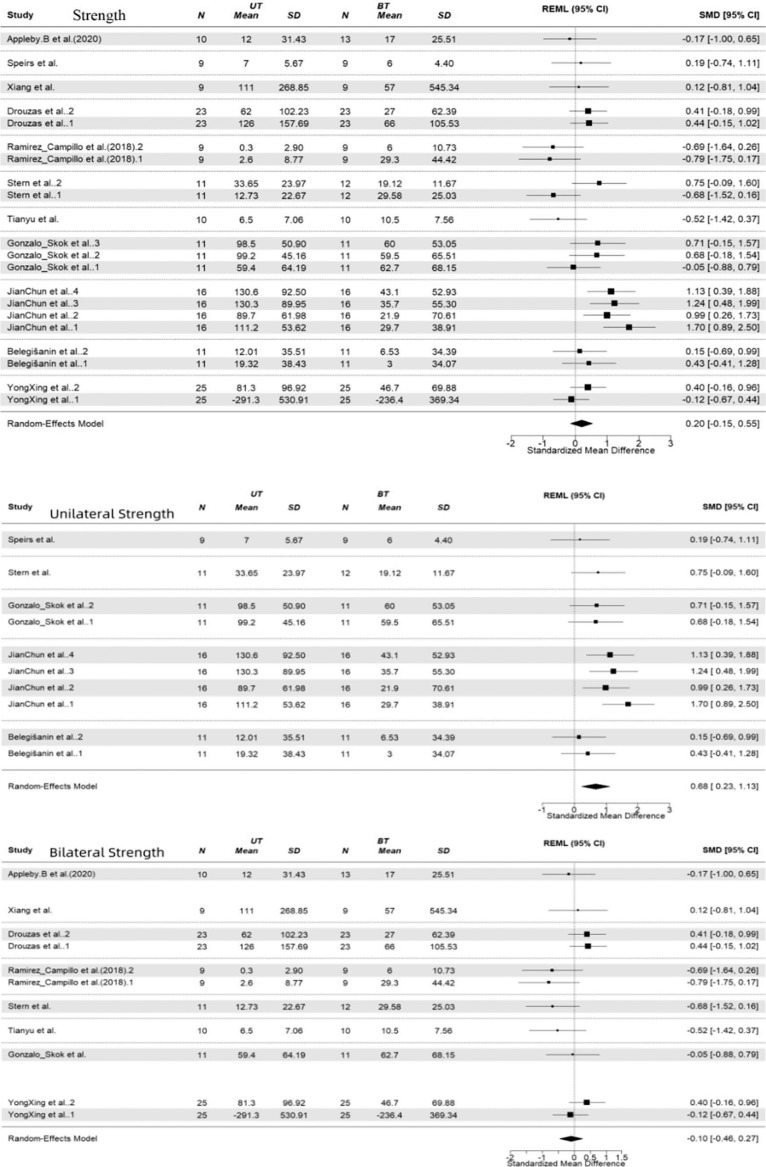
The pooled effect size of the strength. Note: N, the total number of effects included in the pooled effect size; UT, unilateral training; BT, bilateral training; 95%CI, 95% confidence interval; SMD, standardized mean difference; SD, standardized difference.

**FIG. 4:**
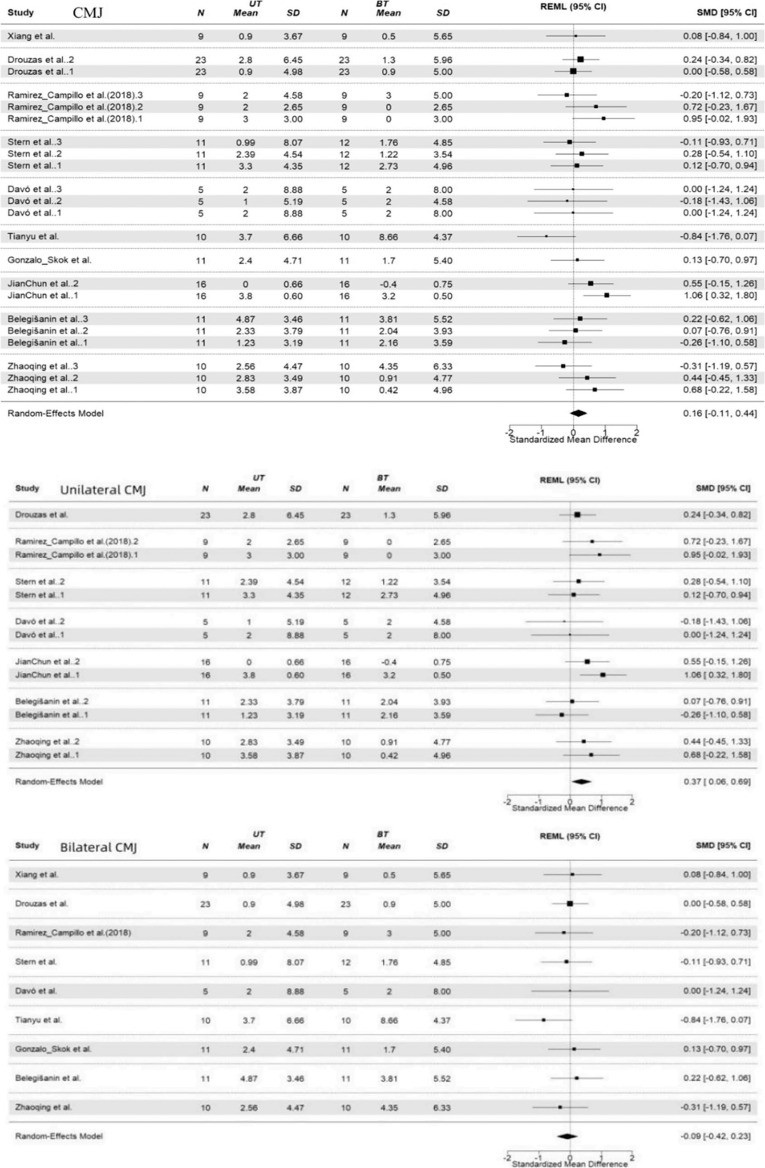
The pooled effect size of the CMJ. Note: N, the total number of effects included in the pooled effect size; UT, unilateral training; BT, bilateral training; 95%CI, 95% confidence interval; SMD, standardized mean difference; SD, standardized difference.

**FIG. 5:**
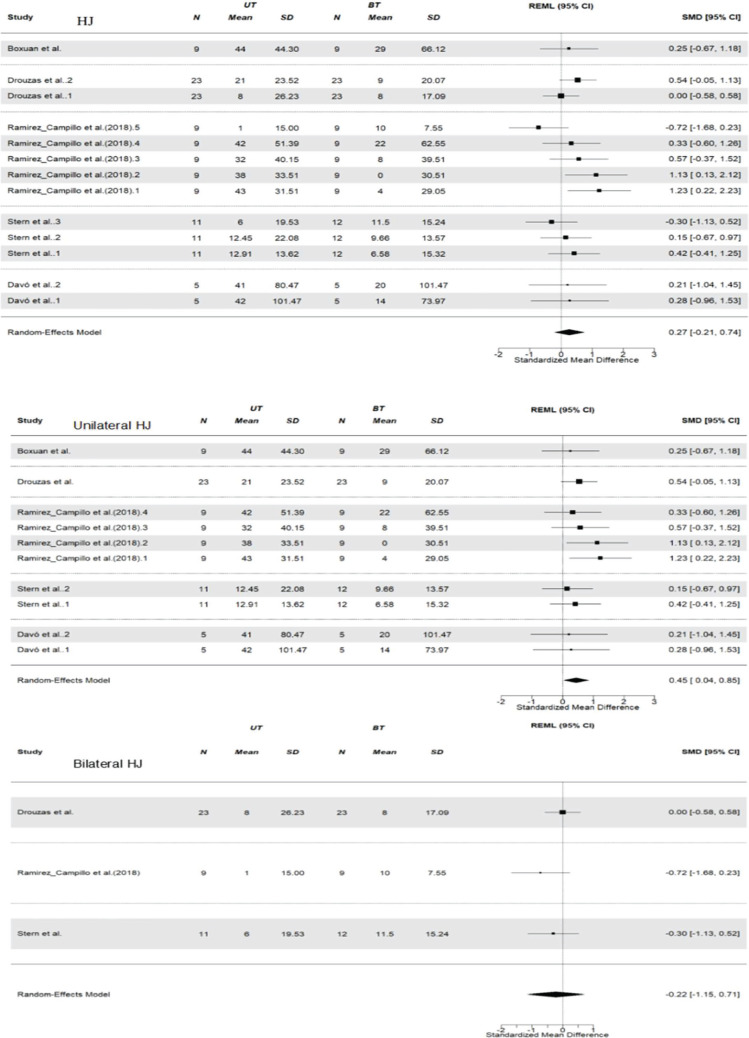
The pooled effect size of the HJ. Note: N, the total number of effects included in the pooled effect size; UT, unilateral training; BT, bilateral training; 95%CI, 95% confidence interval; SMD, standardized mean difference; SD, standardized difference;HJ, horizon jump.

**FIG. 6:**
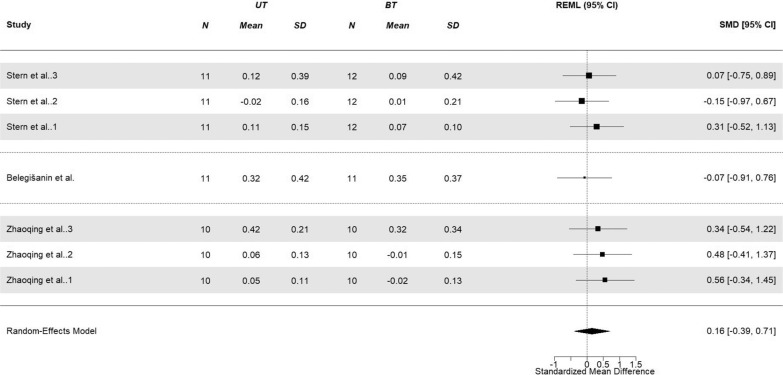
The pooled effect size of the RSI. Note: N, the total number of effects included in the pooled effect size; UT, unilateral training; BT, bilateral training; 95%CI, 95% confidence interval; SMD, standardized mean difference; SD, standardized difference;RSI, reactive strength index.

**FIG. 7:**
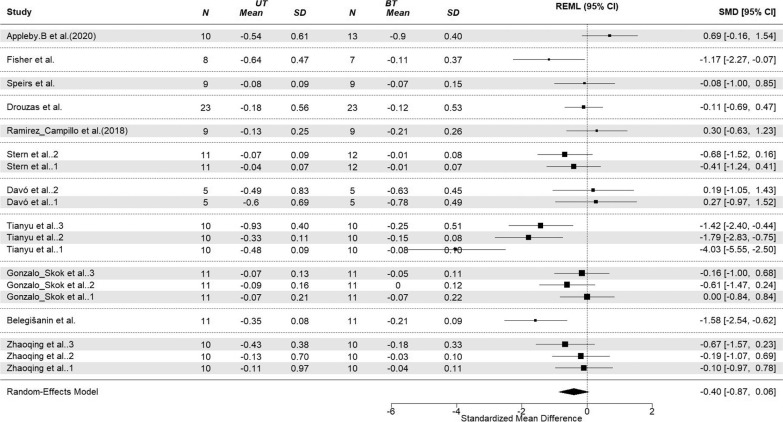
The pooled effect size of the agility. Note: N, total number of effects included in the pooled estimate; UT, unilateral training; BT, bilateral training; SMD, standardized mean difference; SD, standard deviation; 95%CI, 95% confidence interval. Negative SMD values indicate greater reductions in agility time (i.e., better agility performance) in the UT group compared with the BT group, whereas positive values indicate better agility performance in the BT group.

**FIG. 8:**
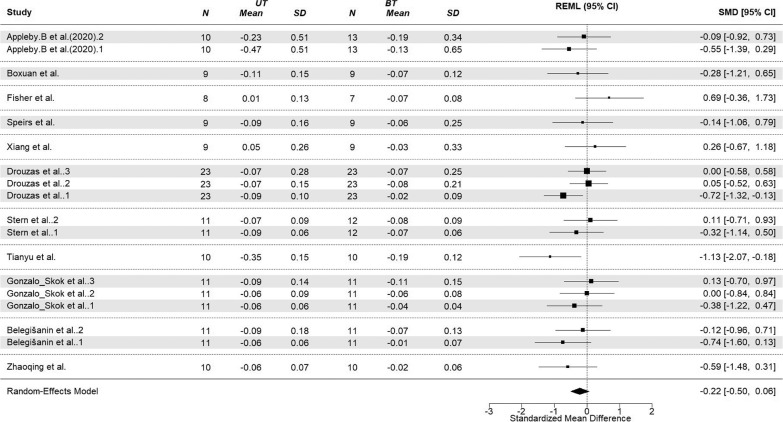
The pooled effect size of the sprint. Note: N, total number of effects included in the pooled estimate; UT, unilateral training; BT, bilateral training; SMD, standardized mean difference; SD, standard deviation; 95%CI, 95% confidence interval. Negative SMD values indicate greater reductions in sprint time (i.e., better sprint performance) in the UT group compared with the BT group, whereas positive values indicate better sprint performance in the BT group.

#### Strength

Eleven studies (21 variables) were included in the strength analysis. No significant difference was found between UT and BT (g = 0.20, 95% CI [-0.15 to 0.55], p = 0.25). However, compared to BT, UT demonstrated a significant and moderate-to-large advantage in unilateral strength (g = 0.68, 95% CI [0.23, 1.13], p = 0.007), while no difference was observed for bilateral strength (g = -0.10, 95% CI [-0.46, 0.27], p = 0.56).

#### Jumping

A total of 10 studies (22 indicators) were included in the CMJ analysis. No significant difference was noted between UT and BT in overall CMJ outcomes (g = 0.16, 95% CI [-0.11, 0.44], p = 0.24). However, UT demonstrated a significant, moderate improvement in unilateral CMJ performance compared to BT (g = 0.37, 95% CI [0.06, 0.69], p = 0.025), while no difference was found in bilateral CMJ (g = -0.09, 95% CI [-0.42, 0.23], p = 0.53).

For the HJ analysis, (5 studies, 13 indicators), there was no significant difference between UT and BT (g = 0.27, 95% CI [-0.21, 0.74], p = 0.25). UT demonstrated a significant, small-to-moderate improvement in unilateral HJ compared to BT (g = 0.45, 95% CI [0.04, 0.85], p = 0.03), with no difference between UT and BT for bilateral HJ (g = -0.22, 95% CI [-1.15, 0.71], p = 0.41).

In the RSI analysis, (3 studies, 7 test measures), neither UT nor BT significantly improved unilateral RSI (g = 0.28, 95% CI [-0.60, 1.15], p = 0.39) or bilateral RSI (g = 0.10, 95% CI [-0.97, 1.17], p = 0.72).

#### Agility

A total of 11 studies (19 indicators) were included in the agility analysis. Results showed no significant difference in agility between UT and BT (g = -0.40, 95% CI [-0.87, 0.06], p = 0.08).

#### Sprinting

A total of 11 studies (18 indicators) were included in the sprint analysis. Results showed no significant difference between UT and BT on sprint performance (g = -0.22, 95% CI [-0.50, 0.06], p = 0.12).

Further details on sensitivity analyses and publication bias assessments for each outcome are provided in Electronic Supplementary Material Appendix S3.

#### Moderator Analysis

A factor analysis was conducted to investigate the effects of sport type and training program on the performance of team sports athletes under unilateral and bilateral interventions.

#### Effects by Sport

Overall, no significant differences were observed between UT and BT across sports (P > 0.05). However, in basketball, compared to other performances, UT led to significant improvements in both agility and sprint performance, with effect sizes ranging from moderate to large (g = -0.77, 95% CI [-1.55, 0.00], p = 0.04; g = -0.37, 95% CI [-0.74, -0.00], p = 0.04).

#### Effects by Intervention Type

No significant differences were found between UT and BT across intervention types (p > 0.05). However, compared to other intervention types, the combination of UT with PT yielded significant improvements in strength (with a moderate effect size, g = 0.54, 95% CI [0.05, 1.03], p = 0.03) and in sprint performance (with a small effect size, g = -0.30, 95% CI [-0.59, -0.02], p = 0.03). Additionally, UT implemented with CT significantly enhanced agility, showing a moderate-to-large effect (g = -0.74, 95% CI [-1.47, -0.02], p = 0.04).

### Risk of Bias and Quality of Methods

The risk of bias for each study and PEDro scale is depicted in Electronic Supplementary Material Appendix S3. Publication bias was assessed using funnel plots and Egger’s test across outcomes of strength, jumping, agility, and sprint performance. The Egger regression analysis indicated potential publication bias for unilateral strength (p < 0.05), bilateral strength (p < 0.01), and agility (p < 0.01), while no significant bias was found for the other nine indicators (p > 0.05). Electronic Supplementary Material Appendix 3.

### Sensitivity Analysis

Leave-one-out sensitivity analyses (Supplement Table S3 of the ESM) showed that excluding any single study did not significantly impact the overall pooled outcomes, confirming the robustness and reliability of our findings, consistent with Kadlec et al [86].To determine the appropriate value of the correlation coefficient (r) between effect sizes in the three-level model, a sensitivity analysis was conducted by examining r values ranging from 0.5 to 0.9. The results indicated that when r = 0.6, the pooled effect size estimate was most stable (showing the smallest deviation from the median), and the heterogeneity structure was reasonably acceptable. Therefore, r = 0.6 was selected for the final model fitting to enhance the robustness and interpretability of the findings. A detailed rationale is provided in Electronic Supplementary Material Appendix S4. Furthermore, the GRADE evidence rating results of this systematic review are presented in Electronic Supplementary Material Appendix S5.

## DISCUSSION

This meta-analysis of 15 studies compared UT and BT effects on lower-limb performance in team sports athletes, focusing on strength, jumping, sprinting and agility. Given the prevalence of asymmetric unilateral movements in these sports, the specificity of UT appears to drive improvements in unilateral performance that can influence the efficacy of on-court movements. UT produced significant improvements in unilateral strength (g = 0.68, p = 0.007), unilateral CMJ (g = 0.37, p = 0.025), and unilateral HJ (g = 0.45, p = 0.03). In contrast, no significant differences were observed between UT and BT for bilateral strength, bilateral jumping, RSI, agility, or sprinting. These findings highlight the unique benefits of UT for unilateral tasks and offer practical guidance for optimizing training in team sports.

### Strength

Although no significant advantage of UT was observed over BT for overall strength (g = 0.20, 95% CI [-0.15, 0.55], p = 0.25) and bilateral strength (g = -0.10, 95% CI [-0.46, 0.27], p = 0.56), compared to BT, UT showed a significant positive effect on unilateral strength (g = 0.68, 95% CI [0.23, 1.13], p = 0.007). The results of the strength investigation indicate that unilateral and bilateral training do not fully conform to the principle of specialized training. This is because we only found a specialized advantage for unilateral strength in the UT group, but did not observe a specialized advantage for bilateral strength in the BT group. UT exercises, such as single-leg squats, replicate unilateral movements and enhance neuromuscular adaptations within one limb, including enhanced motor unit recruitment and intermuscular coordination [79]. Since team sports (e.g., football and basketball) also involve single-leg actions such as shooting and executing a three-step layup, the unilateral benefits of UT are particularly relevant.

The lack of significant bilateral strength effects may reflect the heterogeneous training backgrounds of team-sport athletes. Previous studies indicate that athletes at different training levels exhibit distinct adaptations and bilateral strength imbalances. High-level athletes often demonstrate greater stability and more balanced lower-limb strength, which may limit further bilateral strength improvements [31]. Moreover, frequent bilateral movements in routine technical training could reinforce bilateral strength, yielding comparable outcomes between modalities. Our findings align with Zhang et al. [23] (g = 8.95, 95% CI [2.30, 15.61], p = 0.008) but differ from the bilateral strength advantages reported by Kassiano et al. [30] and Kaifang Liao et al. [29]. These discrepancies may stem from differences in inclusion criteria (e.g., study sources, intervention duration, or training load) or testing methods (e.g., isokinetic vs. dynamic strength assessments) [30]. Among the 15 included studies, the average intervention period was 7.3 weeks, with testing methods encompassing both dynamic and joint isokinetic strength assessments.

Some researchers argue that bilateral strength gains from BT reflect training specificity, where task coordination improves with practice [87, 88]. Häkkinen et al. [89] reported that BT increases surface electromyographic (EMG) activity in both legs more than UT. In contrast, our analysis revealed that UT significantly improved strength when paired with PT (g = 0.54), marking the first evidence of intervention type as a moderator. Compared with other interventions, PT enhances strength by utilizing the stretch-shortening cycle to store elastic potential energy and generate explosive force [23], thereby boosting physical fitness [90]. PT has been shown to significantly improve strength across various sports [91], including team sports disciplines [92]. These gains are attributed to neural adaptations (i.e., increased motor unit discharge frequency, synchronization, and excitability [93]) and muscle hypertrophy [94]. Despite some inconsistencies in the literature, our results reinforce the evidence for the clear advantages of UT in developing unilateral strength.

### Jumping

Analysis of CMJ performance showed no significant differences in overall CMJ (g = 0.16, 95% CI [-0.11, 0.44], p = 0.24) and bilateral CMJ (g = -0.09, 95% CI [-0.42, 0.23], p = 0.53), but unilateral CMJ showed a significant advantage for UT, compared to BT (g = 0.37, 95% CI [0.06, 0.69], p = 0.025). These findings are consistent with previous reviews [23, 29]. Improvements in vertical jump height often coincide with gains in strength, sprint speed, and long-distance running capacity [95–97], all critical to athletic performance [98]. The unilateral CMJ enhancement highlights UT’s benefits for single-leg take-off tasks such as basketball dunks and volleyball spikes. During UT, the reduced base of support and contact area engage synergistic and stabilizing muscles [11], enhancing single-limb explosive power and stability through coordinated hamstrings and quadriceps action [19]. Supporting this, Bogdanis et al. [22] found unilateral jump training improves unilateral force development rate more effectively than bilateral jump training. The lack of bilateral CMJ differences suggests that both UT and BT effectively enhance bilateral vertical jump performance. Furthermore, UT may indirectly enhance bilateral jumping via the cross-education effect, improving performance in the untrained limb [99]. Pending confirmatory research, this cross-education phenomenon could be mediated by neural adaptations within the cerebral cortex, spinal cord, and neuromuscular system, potentially mediated by inhibitory interactions between limbs [23].

Routine technical training may aid bilateral vertical jump development. However, the significant UT advantage reported by Zhang et al. [27] (g = 0.53, 95% CI [0.02, 1.04], p = 0.04) differs from our findings. Subgroup analysis showed that a total ground contact frequency of ≤ 900 repetitions significantly enhanced vertical jump performance. We attribute these discrepancies to differences in vertical jump testing methods, training program design, and participant characteristics. Specifically, Zhang et al. [27] included five squat jump metrics, while our analysis focused solely on CMJ metrics. Moreover, biomechanical differences between slow and fast stretchshortening cycle contractions produce distinct mechanical outputs and jump performances [100, 101]. Accordingly, we recommend that future research standardize both testing methods and jump types to improve comparability.

Analysis of HJ performance showed no significant differences between UT and BT for overall HJ (g = 0.27, 95% CI [-0.21, 0.74], p = 0.25) and bilateral HJ (g = -0.22, 95% CI [-1.15, 0.71], p = 0.41), but compared to BT, UT demonstrated a significant advantage on unilateral HJ (g = 0.45, 95% CI [0.04, 0.85], p = 0.034). This improvement underscores the advantage of UT in tasks requiring single-leg explosive power. Unlike vertical jumps, horizontal jumps place greater emphasis on hip flexion and extension [102] and takeoff angle [103]. Enhancements in unilateral strength from UT likely enhance propulsive force in unilateral HJ. Although vertical jump ability often receives great attention, HJ distance may be equally or more relevant for assessing functional movement and correlates more strongly with sprint performance [31, 104]. The absence of significant bilateral HJ differences suggests that both UT and BT effectively enhance bilateral HJ, potentially influenced by core strength contributions [27]. These findings further highlight the specific benefits of UT for unilateral HJ performance.

Analysis of the RSI (3 studies, 7 indicators) revealed no significant differences between UT and BT for either unilateral RSI (g = 0.28, 95% CI [-0.60, 1.15], p = 0.39) or bilateral RSI (g = 0.10, 95% CI [-0.97, 1.17], p = 0.72). RSI reflects an athlete’s ability to rapidly transition from eccentric to concentric contraction and is calculated by dividing vertical jump height by ground contact time [105]. It is a key metric for explosive jumps in team sports, particularly for fast stretch-shortening cycle actions like jumping. Additionally, RSI moderately correlates with strength, linear speed, endurance, and change of direction performance [106]. Despite UT producing significant unilateral strength gains, no corresponding RSI improvements were observed, contrasting with some previous studies [107, 108]. This may be due to the small number of studies limiting statistical power. Additionally, RSI is influenced by technique, muscle fiber composition, and training specificity, which may obscure differences between training methods [105]. Variations in RSI metrics (e.g., RSI vs. RSImod) and differences in drop heights (e.g, 20–40 cm) can also affect RSI outcomes [109, 110]. Future research should increase sample sizes, standardize testing protocols (e.g., fixed-height drop jumps), and incorporate surface electromyography (EMG) analyses to better understand how UT and BT influence RSI.

### Agility

Analysis of agility performance (11 studies, 19 indicators) revealed no significant difference between UT and BT (g = -0.40, 95% CI [-0.87, 0.06], p = 0.08). Agility is a critical ability in team sports, encompassing multiple components such as technique, reaction time, leg strength, linear speed, and cognitive processing [111]. Overall, results indicate comparable effects of UT and BT on agility. One study on football players found comparable agility improvements regardless of training type via two-way ANOVA [112]. Likewise, Campillo et al. [113] found that while the training group with UT improved significantly compared to controls, no significant difference emerged between the two training groups. Although UT does not significantly enhance agility, some studies indicate a notable advantage for unilateral agility [21, 76]. This is attributed to UT’s ability to enhance neuromuscular adaptations and elastic energy utilization [23, 114], thereby improving unilateral braking and propulsion capabilities [115]. Recent studies also highlight eccentric adaptations as a key mechanism, with eccentric strength and vector-specific force production identified as modifiable factors for change of direction [7, 116]. Additionally, eccentric force during single-leg support exceeds that during double-leg support [73]. Consequently, future research should focus on the effects of unilateral eccentric training on agility. The near-significant result (p = 0.08) indicates a potential effect that merits further investigation with larger sample sizes. This analysis was strengthened by incorporating multiple agility tests to enhance comprehensiveness.

Moderator analyses indicated a statistically significant pooled effect for agility within the basketball subgroup (g = -0.77); however, this finding should be interpreted with caution, given the limited number of studies and the possibility that the result is influenced by one or a few individual trials. These data are better viewed as preliminary and a hypothesis. Basketball’s sport-specific demands, such as frequent rebounding and rapid, multi-directional movements, place high loads on unilateral lower-limb function, which may help explain why UT could be particularly relevant in this context [117, 118]. Consistent with this notion, Tianyu et al [77]. reported that UT improved change-of-direction ability in basketball players and suggested that this benefit may stem from similarities between UT movement patterns and basketball-specific actions (e.g., sliding defense) that promote neuromuscular adaptations related to directional changes, a view supported by several other studies [21, 75, 119]. Further robust research is needed to validate these preliminary exploratory findings. Regarding intervention type, UT utilizing CT demonstrated significant improvements in agility (g = -0.74). CT that combines resistance training and PT is known to effectively enhance athletic performance [79, 80]. Meta-analyses of UT and BT also identify CT as a key driver of muscular performance gains [23]. The agility benefits of CT may arise from RT facilitating center-of-gravity control during direction changes [120, 121], while PT augments muscle recruitment by enhancing elastic energy storage and release. Additionally, countermovement jump exercises effectively develop hamstring strength, further enhancing change-of-direction and agility performance [77, 122].

### Sprint

Analysis of sprint performance (11 studies, 18 indicators) revealed no significant difference between UT and BT (g = -0.22, 95% CI [-0.50, 0.06], p = 0.12). Linear sprinting is a fundamental skill in team sports that is driven by alternating single-leg propulsion. Although UT is often considered more effective for sprinting due to its specific biomechanical specificities [23], our findings suggest that both training methods can improve sprint performance through distinct mechanisms: UT primarily develops single-leg strength, while BT improves overall force production. Moran et al. [123] (g = 0.17, 95% CI [-0.15, 0.50], p = 0.30) reported comparable findings. Evidence indicates that UT facilitates linear sprint development via enhanced neuromuscular activation [23] and potentially reduces bilateral force deficits [123]. Both UT and BT can also increase core muscle engagement through induced instability [124, 125], while BT provides the additional advantage of accommodating heavier external loads [126]. Furthermore, studies on elite athletes suggest BT may derive greater benefits [127], highlighting unique advantages for each training method. Discrepancies with previous reviews may stem from differences in sprint test distances (e.g., 10-m vs. 30-m sprints), as these capture distinct physical qualities [128].

A statistically significant pooled effect for sprint performance was observed within the basketball subgroup (g = -0.37); however, this result should be interpreted cautiously, as the number of available studies is limited and the estimate may be influenced by one or a few individual trials rather than reflecting a robust sport-specific effect. In basketball, frequent short sprints are integral to decisive offensive and defensive actions, which may partly explain why UT appears particularly relevant in this context [129, 130]. Duan et al [77]. reported greater sprint improvements with UT in basketball players and attributed this to training specificity, namely, closer matching of the muscle activation patterns and neuromuscular coordination demands of sport-specific movements. With respect to intervention type, plyometric training within UT programs showed a significant advantage for sprint performance (g = -0.30), again warranting cautious interpretation given subgroup sample sizes. Previous work has identified PT as a particularly effective UT modality for enhancing horizontal sprinting [31], and both horizontal and vertical PT have been shown to improve sprint performance [31]. From a contact-time perspective, PT closely mimics the brief ground-contact times characteristic of sprinting [123], and its emphasis on vertical and lateral movements, large joint ranges of motion, and stretch–shortening cycle contractions may underlie these benefits [131]. Nevertheless, the current findings should be viewed as preliminary and hypothesis-generating, and further well-designed trials across different sports are needed before firm conclusions can be drawn about sport- or modality-specific sprint advantages with UT.

Overall, compared with BT, UT aligns more closely with sportspecific training principles in team sports and demonstrates superior transfer to lower-limb performance. UT offers a clear advantage in unilateral explosiveness while delivering comparable outcomes to BT in other performance domains. Both training methods effectively engage stabilizing and synergistic muscles and enhance neuromuscular coordination [11, 107, 132]. Moreover, the cross-transfer effect of UT on the untrained limb offers a versatile training option. Its lower limb loading also makes UT preferable for athletes with back pain or those recovering from injury. However, excessive reliance on UT may impair movement technique, posture, and strength development [30, 133, 134]. Therefore, training should avoid overreliance on a single method and instead combine UT and BT based on the athlete’s training status and performance objectives. Subgroup analysis further revealed that the effects of UT and BT on lower-limb performance were moderated by sport and intervention type, with significant improvements observed in PT and CT interventions. Sport type was not identified as a significant moderator; however, in basketball, but not in other sport types, UT produced greater improvements in agility and sprint performance. We recommend that future studies validate these findings through longitudinal studies and include female cohorts.

### Research significance and impact

This study provides valuable guidance for team sports training. The clear advantages of UT in enhancing unilateral strength and jumping indicate that it should be a core component of team-sports training programs, particularly to improve single-leg performance, such as single-leg take-offs and changes of direction. The comparable effects of UT and BT on bilateral tasks and other performance indicators offer coaches the flexibility to integrate training methods according to sport-specific demands; for example, emphasizing UT during skillfocused phases and BT during strength-accumulation phases. Theoretically, these findings support training specificity and provide indirect evidence of neuromuscular adaptations underlying both methods. Practically, optimizing training design may enhance competitive performance and reduce injury risk by improving unilateral strength and stability. Future research should investigate the role of UT in injury prevention and determine the optimal combination of training modalities.

### Potential limitations and future direction

This study has several limitations. First, the small number of included studies on RSI (n = 3) limits statistical power and generalizability. Second, heterogeneity in training protocols, testing methods, and participant characteristics (e.g., intervention periods ranging from 6 to 12 weeks) may have affected result robustness. Most studies involved young athletes, with limited or no data on adults, highly trained athletes, and women, restricting the generalizability of our findings. In addition, the subgroup results reported in this meta-analysis, particularly those suggesting sport- or modality-specific results, should be regarded as preliminary and exploratory and interpreted with caution until they are confirmed by larger, well-designed trials.

Our findings demonstrate that discrepancies exist in both testing methodologies and procedural protocols across several fundamental assessments, which has partially augmented the heterogeneity of existing research. Consequently, we recommend that well-established gold-standard measures—such as unilateral and bilateral jump tests and agility assessments—should be standardized and explicitly defined in future studies. Furthermore, our research reveals that within studies investigating the effects of unilateral versus bilateral training on team sport athletes, the majority of outcome measures focus on muscular strength and jump performance. In contrast, relatively few studies have examined the impacts of such training on agility, sprint performance, or even cardio-respiratory function. We therefore propose that future research endeavors should prioritize and expand empirical exploration into these understudied domains. Finally, regarding research on unilateral and bilateral training in team sports, the study population remains predominantly concentrated on adolescent male athletes. To comprehensively validate the applicability of unilateral and bilateral training interventions, future research should significantly enhance efforts targeting underrepresented groups, including female athletes, adult athletes, and clinical rehabilitation populations.

## CONCLUSIONS

This meta-analysis demonstrates that UT confers significant advantages for unilateral strength and unilateral jumping performance in team sports athletes, while producing outcomes comparable to BT for bilateral strength, bilateral jumping, RSI, sprinting, and agility. These findings support the optimization of team-sport training programs by emphasizing UT to improve unilateral movement performance. Future research should investigate the long-term effects, injury prevention potential, and underlying neuromuscular mechanisms of UT and BT to guide evidence-based training practices.

## Supplementary Material

Effects of unilateral and bilateral training on performance in team sports athletes: a systematic review and meta-analysis
