# Supplementary material for: Effects of unilateral and bilateral training on performance in team sports athletes: a systematic review and meta-analysis
Source: Biol Sport. 2026 Mar 16;43:1019–39. doi: 10.5114/biolsport.2026.159564 (PMC13343283; doi:10.5114/biolsport.2026.159564)
Supplement: Effects of unilateral and bilateral training on performance in team sports athletes: a systematic review and meta-analysis [file JBS-43-57603-s1.pdf]

SUPPLEMENTARY MATERIAL APPENDIX S1. Continue

Section and Topic	Item #	Checklist item	Location where item is reported
Certainty assessment	15	Describe any methods used to assess certainty (or confidence) in the body of evidence for an outcome.	6
		**RESULTS**	
Study selection	16a	Describe the results of the search and selection process, from the number of records identified in the search to the number of studies included in the review, ideally using a flow diagram.	6
	16b	Cite studies that might appear to meet the inclusion criteria, but which were excluded, and explain why they were excluded.	6
Study characteristics	17	Cite each included study and present its characteristics.	7
Risk of bias in studies	18	Present assessments of risk of bias for each included study.	12
Results of individual studies	19	For all outcomes, present, for each study: (a) summary statistics for each group (where appropriate) and (b) an effect estimate and its precision (e.g. confidence/credible interval), ideally using structured tables or plots.	7–11
Results of syntheses	20a	For each synthesis, briefly summarise the characteristics and risk of bias among contributing studies.	7–11
	20b	Present results of all statistical syntheses conducted. If meta-analysis was done, present for each the summary estimate and its precision (e.g. confidence/credible interval) and measures of statistical heterogeneity. If comparing groups, describe the direction of the effect.	7–11
	20c	Present results of all investigations of possible causes of heterogeneity among study results.	7–11
	20d	Present results of all sensitivity analyses conducted to assess the robustness of the synthesized results.	7–11
Reporting biases	21	Present assessments of risk of bias due to missing results (arising from reporting biases) for each synthesis assessed.	12
Certainty of evidence	22	Present assessments of certainty (or confidence) in the body of evidence for each outcome assessed.	13
		**DISCUSSION**	
Discussion	23a	Provide a general interpretation of the results in the context of other evidence.	13–17
	23b	Discuss any limitations of the evidence included in the review.	13–17
	23c	Discuss any limitations of the review processes used.	13–17
	23d	Discuss implications of the results for practice, policy, and future research.	13–17
		**OTHER INFORMATION**	
Registration and protocol	24a	Provide registration information for the review, including register name and registration number, or state that the review was not registered.	3
	24b	Indicate where the review protocol can be accessed, or state that a protocol was not prepared.	n/a
	24c	Describe and explain any amendments to information provided at registration or in the protocol.	n/a
Support	25	Describe sources of financial or non-financial support for the review, and the role of the funders or sponsors in the review.	n/a
Competing interests	26	Declare any competing interests of review authors.	n/a
Availability of data, code and other materials	27	Report which of the following are publicly available and where they can be found: template data collection forms; data extracted from included studies; data used for all analyses; analytic code; any other materials used in the review.	n/a

From: Page MJ, McKenzie JE, Bossuyt PM, Boutron I, Hoffmann TC, Mulrow CD, et al. The PRISMA 2020 statement: an updated guideline for reporting systematic reviews. BMJ 2021;372:n71. doi: 10.1136/bmj.n71. This work is licensed under CC BY 4.0. To view a copy of this license, visit https://creativecommons.org/licenses/by/4.0/

Supplementary Material Appendix S2. Database Search Form	
**Search items**	**Content**
**Data source**	PubMed, Google Scholar, Web of Science, CNKI, Proquest, Wan Fang
**Retrieval format**	**"Unilateral training"** ("Unilateral exercises" OR "Unilateral resistance training" OR "Single leg training" OR "Unilateral limb exercises")**"Bilateral training"** ("Bilateral exercises" OR "Bilateral resistance training" OR "Bilateral limb exercises") **Unilateral training and Bilateral training** ("Unilateral and Bilateral resistance training" OR "Unilateral and Bilateral plyometric training" OR "Unilateral and Bilateral Composite/Complex/Combine training" OR "Unilateral and Bilateral flywheel training") **Athletic Performance** ("Jump of ability" OR "Ability of sprint" OR "Maximum force" OR "Ability of change of direction" OR "Ability of Balance") **Collective Ball Sport Athletes** ("Basketball players" OR "Soccer players" OR "Rugby players" OR "Volleyball players" OR "Handball players" OR "Hockey players")
**Language of literature**	English, Chinese
**Type of literature**	Journal, Thesis

S3 TABLE 1. PEDro scale.

	Study	PEDro score
Basketball	Gonzalo-Skok [21], 2017	5
	Bogdan Belegišanin [75], 2025	6
	JianChun [76], 2024	7
	Hernández-Davó [78], 2018	6
	Tianyu [77], 2024	6
	YongXing [74], 2024	7
	Zhaoqing [73], 2021	5
Soccer	Stern [79], 2020	5
	Ramirez-Campillo [80], 2018	6
	Drouzas [81], 2020	4
Rugby	Xiang [82], 2023	5
	Appleby.B [83], 2020	6
	Speirs [84], 2016	6
	Fisher [85], 2014	5
Hockey	Boxuan [72], 2020	4

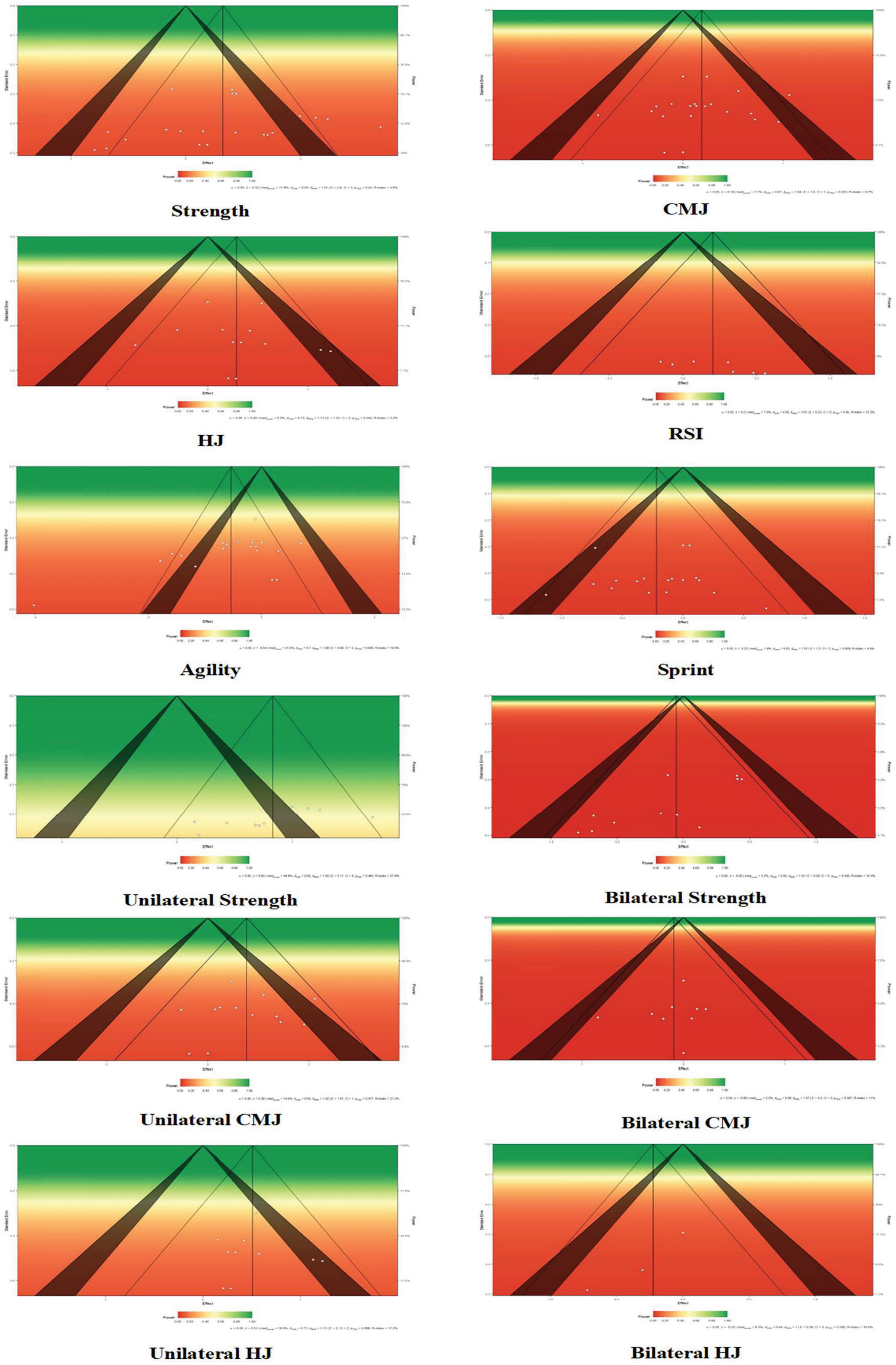

S3 FIG. 1. Funnel plots of each indicator.

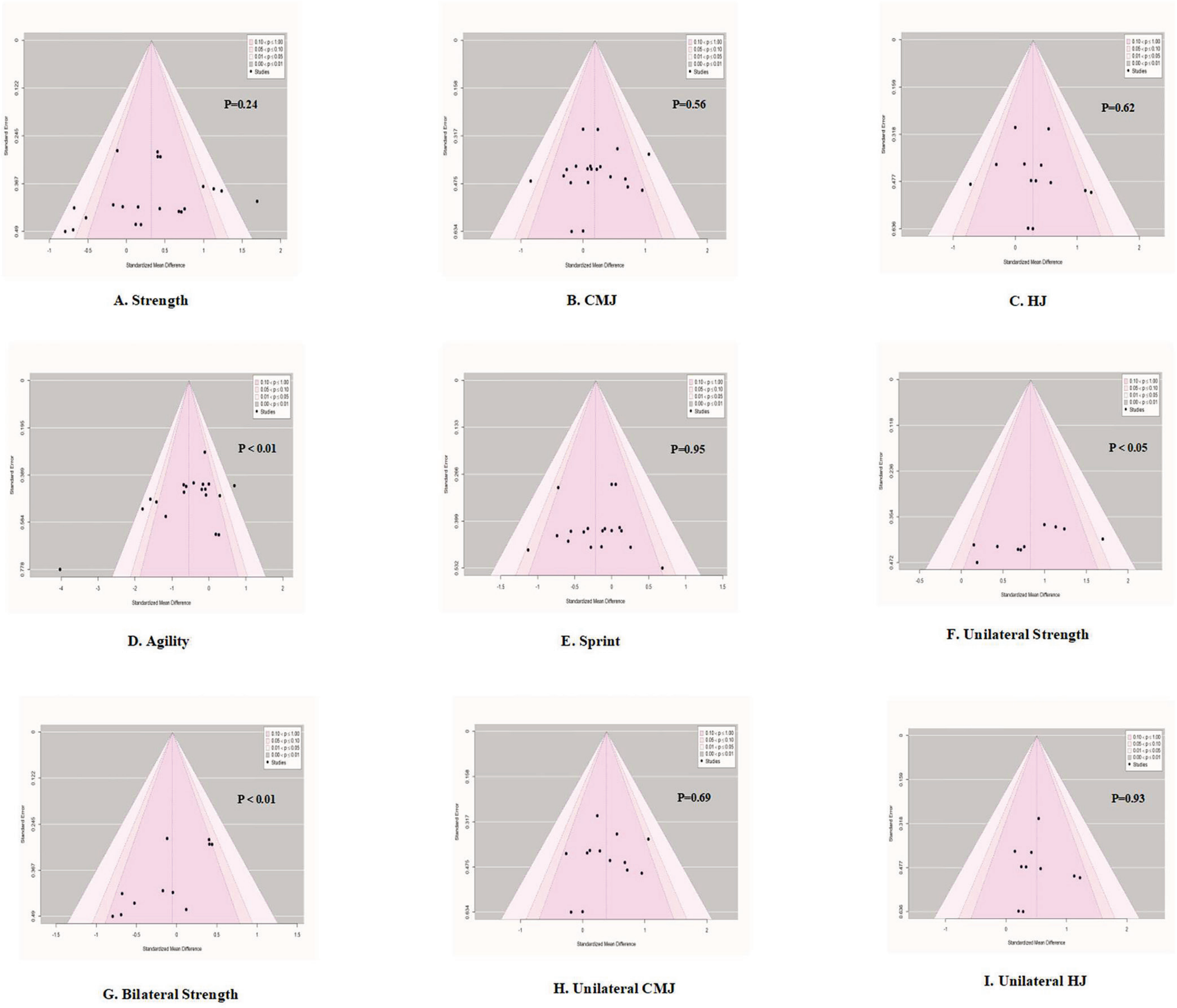

S3 FIG. 2. Publication bias of each indicator.

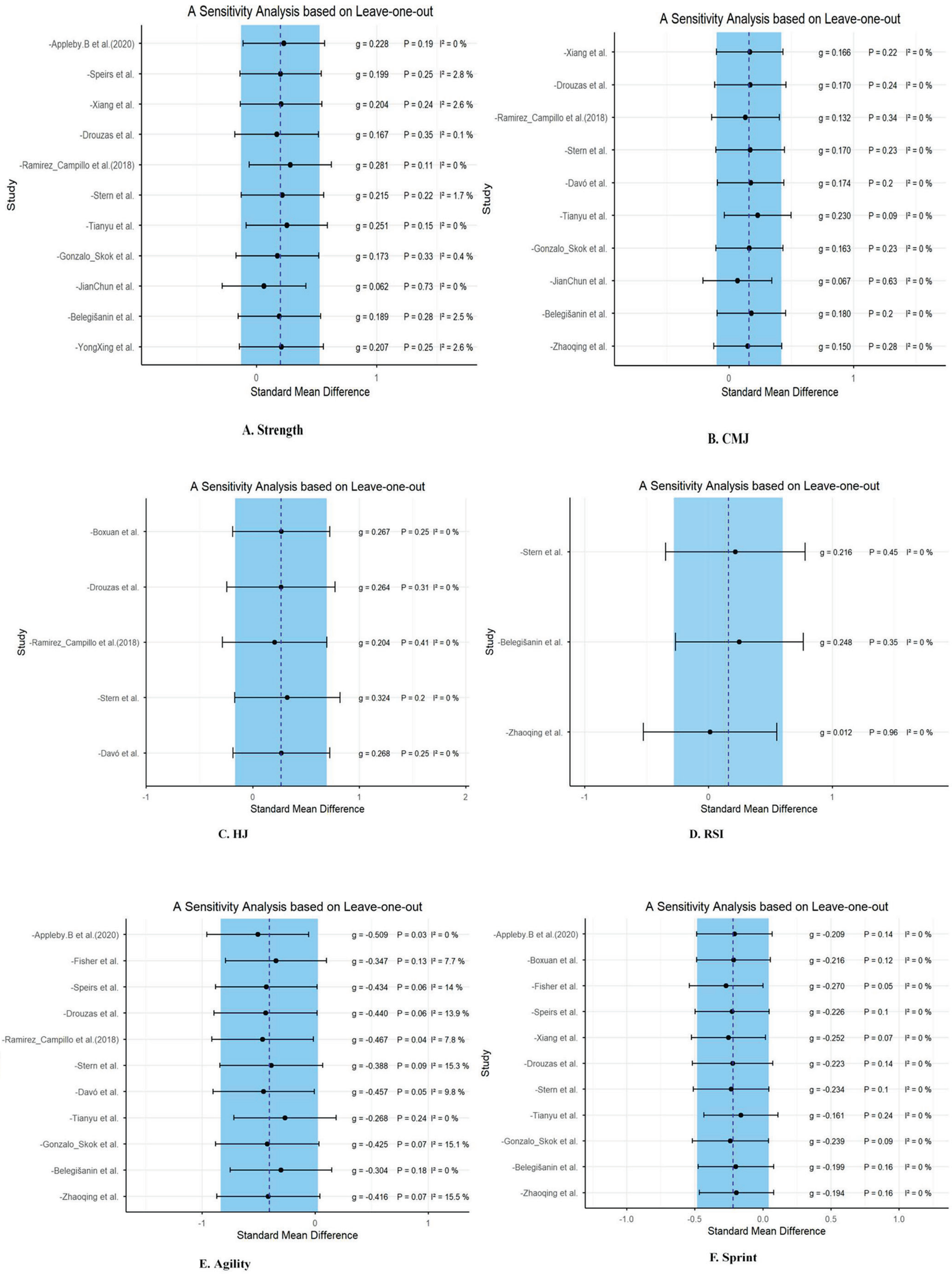

S3 FIG. 3. Sensitivity analysis of each indicator.

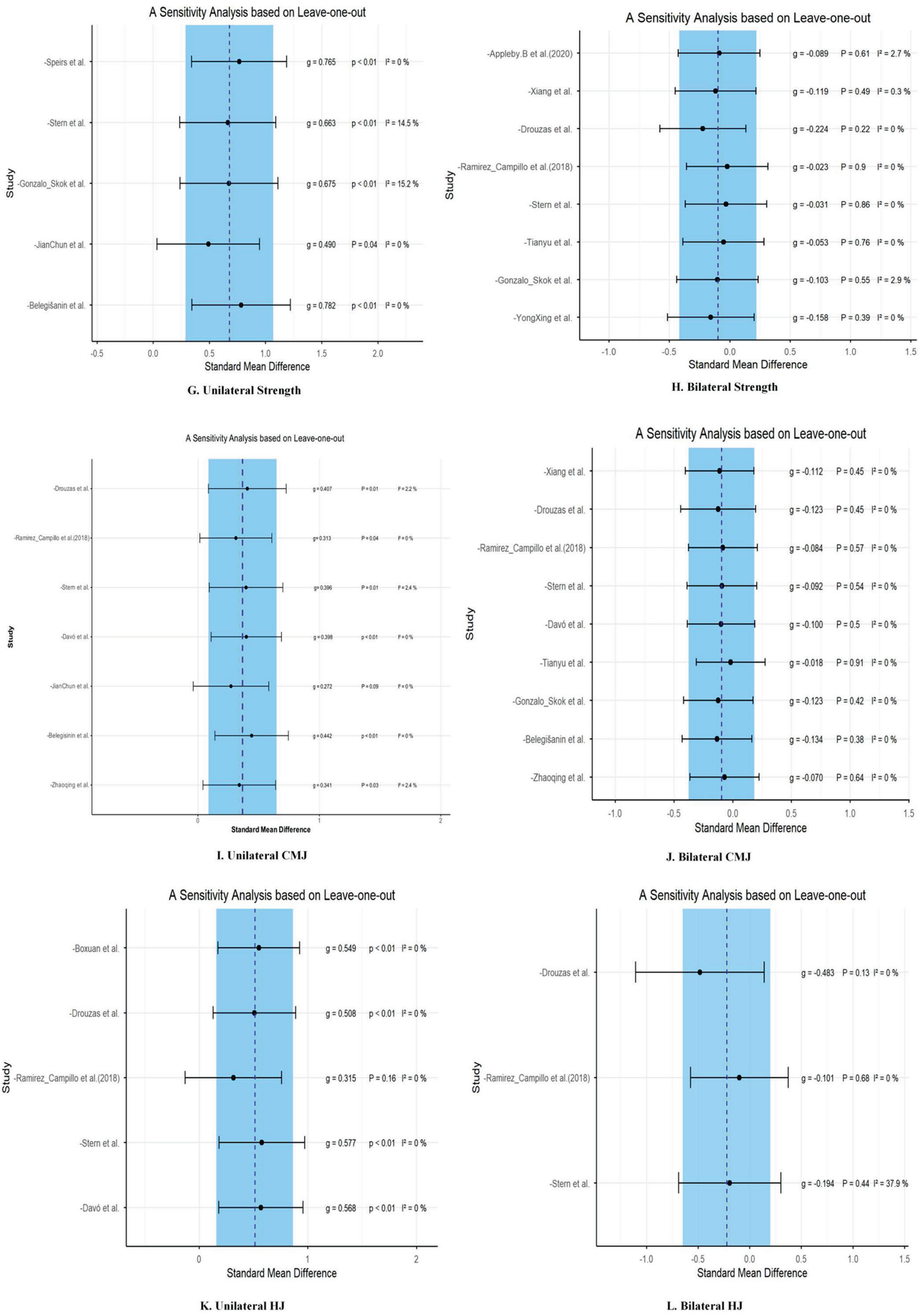

S3 FIG. 4. Sensitivity analysis of each indicator.

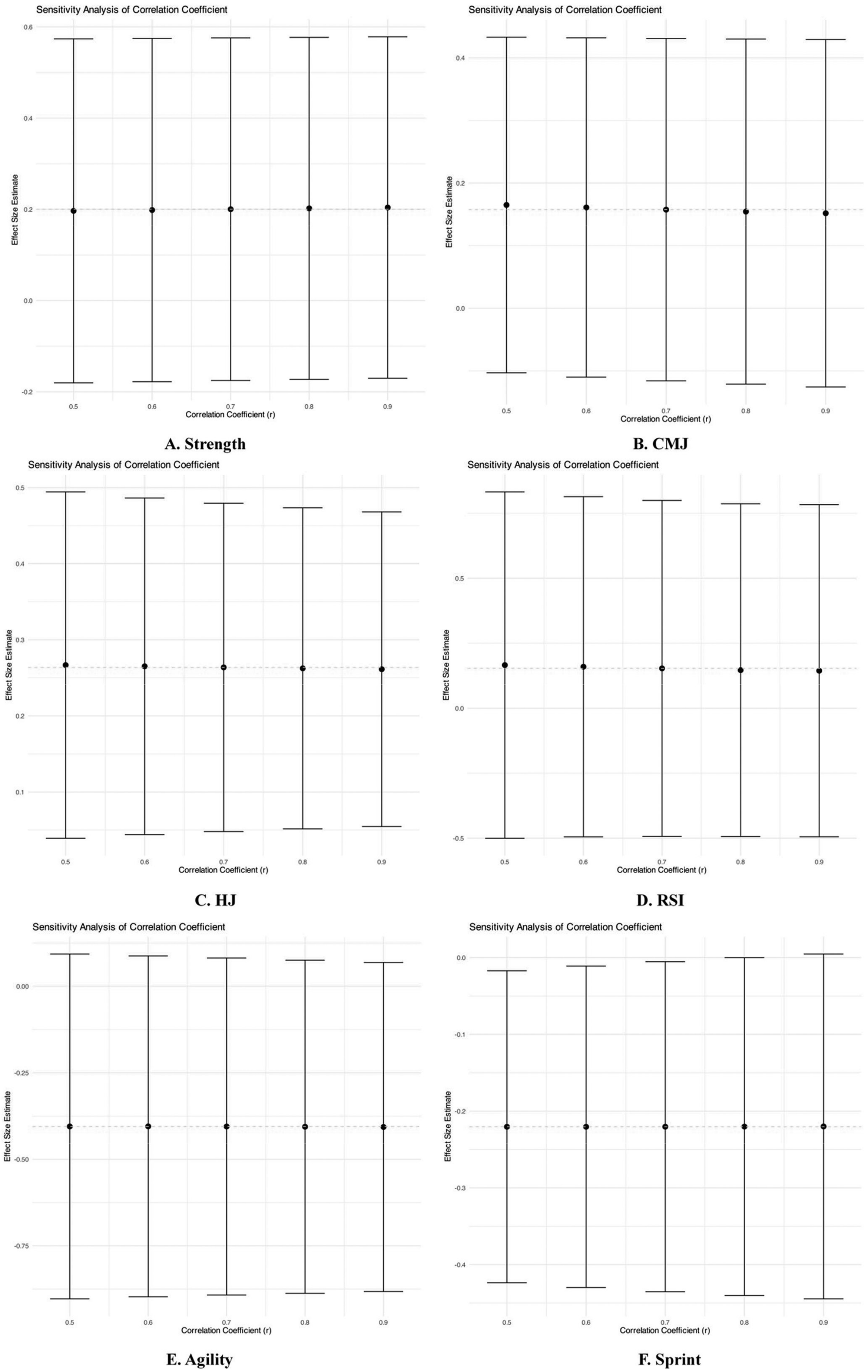

S4 FIG. 1. A sensitivity analysis based on selection R.

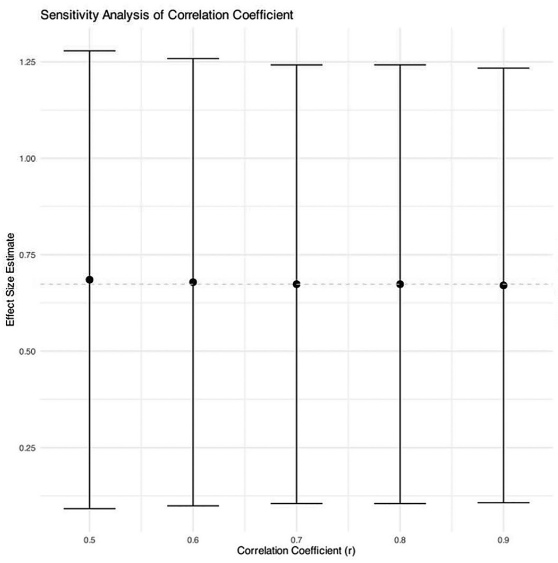

G. Unilatral Strength

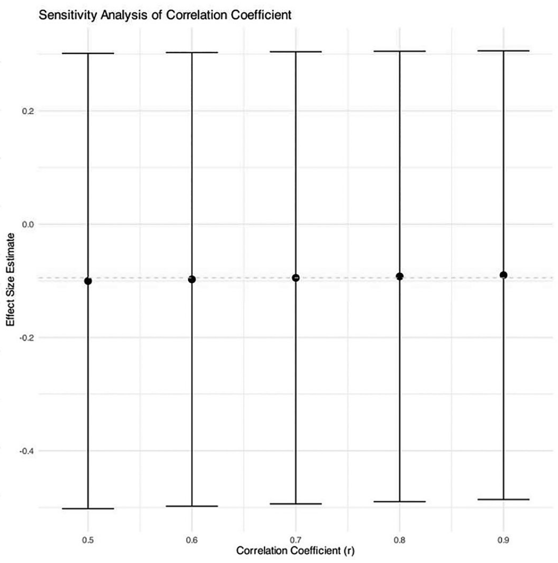

H. Bilateral Strength

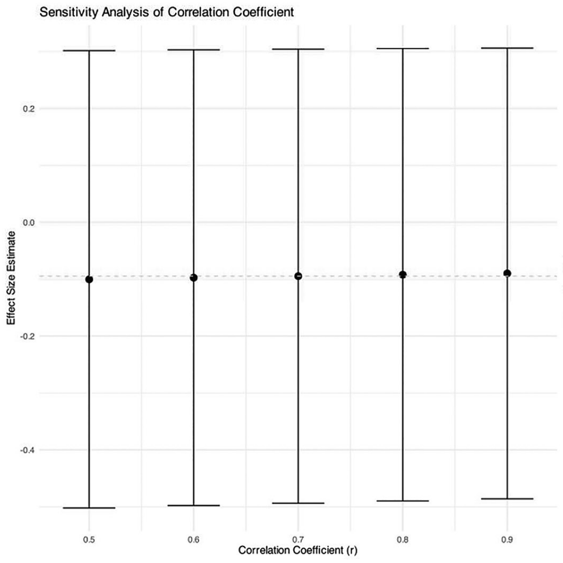

I. Unilateral CMJ

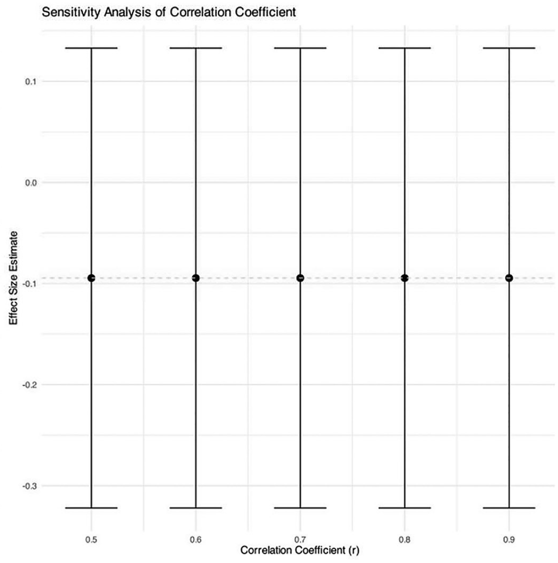

J. Bilateral CMJ

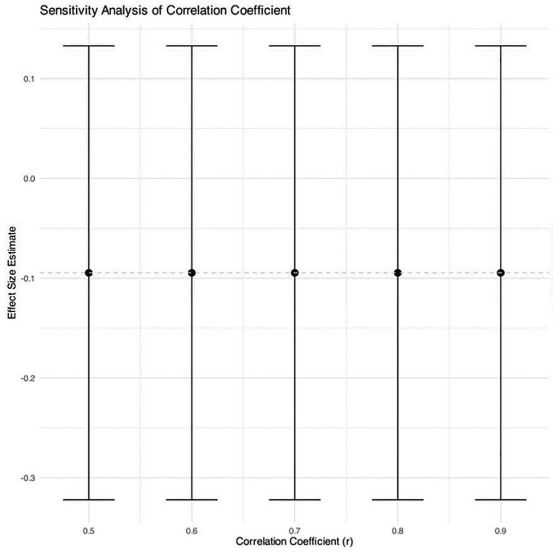

K.Unilateral HJ

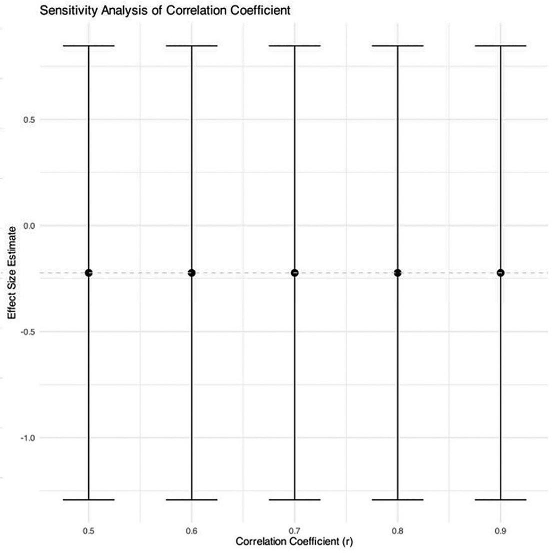

L. BilateralHJ

S4 FIG. 2. A sensitivity analysis based on selection R.

S5 Table 1. GRADE Evidence Certainty Assessment.

Outcome	Participants (RCTs)	GRADE Evidence Certainty Assessment					Hedge's *g* (95% CI)	Certainty*
		Risk of bias	Inconsistency	Indirectness	Imprecision	Other concerns		
**Unilateral Training vs Bilateral Training**								
Unilateral Strength	117 (5 RCT)	Serious	Not serious	Not serious	Serious	Publication bias suspected	0.68 (0.23 to 1.13)	⊕◯◯◯ Very low
Unilateral HJ	115 (5 RCT)	Serious	Not serious	Not serious	Serious	None	0.45 (0.04 to 0.85)	⊕⊕◯◯ Low
Unilateral CMJ	171 (7 RCT)	Serious	Not serious	Not serious	Serious	None	0.37 (0.06 to 0.69)	⊕⊕◯◯ Low
Strength	274 (11 RCT)	Serious	Not serious	Not serious	Serious	None	0.20 (-0.15 to 0.55)	⊕⊕⊕◯ Moderate
Sprint	245 (11 RCT)	Serious	Not serious	Not serious	Serious	None	-0.22 (-0.50 to 0.06)	⊕⊕⊕◯ Moderate
RSI	65 (3 RCT)	Serious	Not serious	Not serious	Serious	None	0.16 (-0.39 to 0.71)	⊕◯◯◯ Very low
HJ	115 (5 RCT)	Serious	Not serious	Not serious	Serious	None	0.27 (-0.21 to 0.74)	⊕⊕◯◯ Low
CMJ	231 (10 RCT)	Serious	Not serious	Not serious	Serious	None	0.16 (-0.11 to 0.44)	⊕⊕⊕◯ Moderate
Bilateral Strength	220 (8 RCT)	Serious	Not serious	Not serious	Serious	Publication bias suspected	-0.10 (-0.46 to 0.27)	⊕◯◯◯ Very low
Bilateral HJ	87 (3 RCT)	Serious	Not serious	Not serious	Serious	None	-0.22 (-1.15 to 0.71)	⊕◯◯◯ Very low
Bilateral CMJ	199 (9 RCT)	Serious	Not serious	Not serious	Serious	None	-0.09 (-0.42 to 0.23)	⊕⊕◯◯ Low
Agility	237 (11 RCT)	Serious	Not serious	Not serious	Serious	Publication bias suspected	-0.40 (-0.87 to 0.06)	⊕◯◯◯ Very low

† GRADE levels of certainty
High: We are very confident that the true effect lies close to the estimate.
Moderate: The true effect is likely close to the estimate, but there is a possibility it is substantially different.
Low: Our confidence in the effect estimate is limited—the true effect may be substantially different.
Very low: We have very little confidence in the effect estimate.

Articles published in the Biology of Sport are licensed under an open access Creative Commons CC BY 4.0 license.
